# Recombinant Protein-Based ELISA for the Detection and Differentiation of Antibodies Against Fowl Adenovirus Serotype 4 in Infected and Vaccinated Chickens

**DOI:** 10.3390/microorganisms14040842

**Published:** 2026-04-08

**Authors:** You Wei, Xiaoqian Wu, Xiaofeng Li, Jiaoling Huang, Bingyi Yang, Liji Xie, Meng Li, Sheng Wang, Aiqiong Wu, Zhihua Ruan, Zhixun Xie, Sisi Luo

**Affiliations:** 1 Guangxi Key Laboratory of Veterinary Biotechnology, Guangxi Veterinary Research Institute, Nanning 530001, China; weiyou0909@163.com (Y.W.); wxx2267464480@163.com (X.W.); lixiaofeng2003@126.com (X.L.); huangjiaoling728@126.com (J.H.); yangby1999@126.com (B.Y.); xie3120371@163.com (L.X.); mengli4836@163.com (M.L.); wangsheng1021@126.com (S.W.); wuaiqiong88@163.com (A.W.); ruanzhihua2020@163.com (Z.R.); 2 Key Laboratory of China (Guangxi)—ASEAN Cross-Border Animal Disease Prevention and Control, Ministry of Agriculture and Rural Affairs of China, Guangxi Veterinary Research Institute, Nanning 530001, China

**Keywords:** fowl adenovirus serotype 4, discriminatory ELISA, infection-derived antibodies, vaccine-induced antibodies, DIVA

## Abstract

Fowl adenovirus serotype 4 (FAdV-4) has been identified as the primary pathogen responsible for hydropericardium-hepatitis syndrome (HHS), resulting in significant economic losses in major poultry-producing countries since 2015. Timely and accurate diagnosis of FAdV-4 infection is essential for the effective prevention and control of HHS. In this study, the two nonstructural genes of FAdV-4, *100K* and *22K*, were inserted into the expression vector pET-32a (+) respectively. The expressed recombinant proteins were used as coating antigens to develop two indirect ELISA methods, designated as 100K-ELISA and 22K-ELISA. Both ELISAs demonstrated high specificity, showing no cross-reactivity with serum samples positive for other avian diseases. Both ELISAs yielded positive results when applied to 50 serum samples from SPF chickens experimentally infected with FAdV-4 and negative results when applied to 50 serum samples from SPF chickens immunized with an inactivated FAdV-4 vaccine. Similarly, the field sample testing results demonstrated a significant ability to distinguish between vaccinated and infected samples. The 100K-ELISA and 22K-ELISA, which are based on nonstructural proteins, may be effective tools for differentiating between FAdV-4 infection and vaccination, offering a promising approach for differentiating infected from vaccinated animals (DIVA) strategies in poultry.

## 1. Introduction

Fowl adenoviruses (FAdVs) belong to the genus Aviadenovirus in the family Adenoviridae. These viruses are classified into 12 serotypes on the basis of serum neutralization assays [1]. In addition, restriction endonuclease analysis of viral DNA enables their classification into five species (FAdV-A to FAdV-E) [2,3]. Among these, FAdV-4 belongs to the species FAdV-C and has been identified as the primary causative agent of hydropericardium-hepatitis syndrome (HHS). Since its emergence in 2015, FAdV-4 has spread extensively across major poultry-producing countries [4]. The virus infects a wide range of avian hosts, including broilers, layers, ducks, geese, pigeons, and mandarin ducks, with young chickens aged 3 to 6 weeks being particularly susceptible [4,5,6]. Mortality rates in infected flocks range from 14% to 80%, depending on factors such as chicken age [5,7], virus strain virulence [8], and coinfection with other pathogens [5], resulting in substantial economic losses [9,10,11].

Timely and accurate diagnosis of FAdV infections is critical for the prevention and control of HHS. Among the available diagnostic methods, the enzyme-linked immunosorbent assay (ELISA) is widely used because of its simplicity, rapidity, high sensitivity, and specificity, making it suitable for large-scale clinical detection. Several studies have reported the development of ELISA methods using purified FAdV virions as coating antigens to detect FAdV antibodies in poultry flocks. Pan et al. [12] developed an ELISA based on highly purified and concentrated FAdV-4 viral particles, which was capable of detecting antibodies against all 12 serotypes and demonstrated enhanced sensitivity to newly isolated Chinese strains of FAdV-4. Similarly, Liu et al. [13] established an indirect ELISA using a purified FAdV-4 strain prevalent in China as the antigen to specifically detect FAdV-4 antibodies. In addition to whole-virus antigens, recombinant protein-based ELISAs have garnered increasing attention. Feichtner et al. [14] developed an indirect ELISA for detecting FAdV-1 antibodies using the recombinant Fiber-1 protein of FAdV-1 as the coating antigen. He et al. [15] expressed the Fiber-2 protein of FAdV-4 to establish an ELISA for detecting FAdV-4-specific antibodies. Furthermore, in addition to antibody detection, Shao et al. [16] developed a sandwich ELISA using two monoclonal antibodies (4A3 and 3C2) targeting the Fiber-2 protein of FAdV-4 to specifically detect FAdV-4 antigens.

With the widespread application of inactivated FAdV-4 vaccines, a new challenge has emerged: distinguishing between antibodies generated by vaccination and those induced by natural infection. After the virus infects the host, it replicates extensively within the body, stimulating the immune system to produce large amounts of antibodies against both structural and nonstructural proteins [17]. Inactivated vaccines remove most nonstructural proteins during processing, leaving only the virus particles. As a result, animals immunized with inactivated vaccines produce few to no antibodies against nonstructural proteins [18]. By detecting antibodies against nonstructural proteins, it is possible to determine whether the antibodies in chickens are generated by infection or by vaccination with inactivated vaccines [17,19]. This theory was first applied in antibody diagnostic differentiation for foot-and-mouth disease virus (FMDV). Studies have shown that the 3ABC nonstructural protein of FMDV could serve as a diagnostic antigen to distinguish antibodies generated by vaccination from those produced by wild-type virus infection. On this basis, diagnostic kits have been developed that have been successfully applied [20]. Currently, both domestically and internationally, nonstructural proteins have been used as diagnostic antigens to develop methods for distinguishing antibodies produced by viral infection from those generated by inactivated vaccine immunization. For example, Girl et al. [21] used recombinant expressed nonstructural protein 1 (NS1) of tick-borne encephalitis virus (TBEV) as a diagnostic antigen to establish an ELISA method capable of distinguishing between TBEV infection and TBE vaccine-induced antibodies.

Wang et al. [22] reported that the nonstructural protein 3A of FMDV can be recognized by the serum of pigs and cattle infected with FMDV but does not react with the serum of unvaccinated or vaccinated healthy animals. Furthermore, Wang et al. [23] constructed recombinant nonstructural proteins from an attenuated strain of porcine reproductive and respiratory syndrome virus (PRRSV) and developed an indirect ELISA method suitable for distinguishing between animals infected with the wild-type strain and those immunized with inactivated vaccines. Xie et al. [24] expressed and purified the nonstructural protein P17 of avian reovirus (ARV) and used it as a coating antigen in an indirect ELISA method, which can distinguish between chickens infected with live ARV and those immunized with inactivated ARV. Huang et al. [19] expressed and purified the NS3 protein of bluetongue virus (BTV) using a prokaryotic expression system and used this recombinant protein as a coating antigen to establish an indirect ELISA method that can differentiate between natural BTV infection and vaccine-induced immunity. Xie et al. [25] expressed the 33K nonstructural protein of the FAdV-1 CELO strain and developed a discriminatory ELISA method capable of distinguishing between FAdV-1-infected chickens and those immunized with inactivated FAdV.

In this study, recombinant plasmids expressing the 100K and 22K proteins of FAdV-4 were constructed, and the corresponding recombinant proteins were used as diagnostic antigens. An ELISA-based serological assay was established to differentiate between vaccine-induced and infection-induced antibodies for DIVA (differentiating infected from vaccinated animals). This method provides a valuable diagnostic tool for the surveillance and eradication of FAdV-4 infections in poultry flocks.

## 2. Materials and Methods

### 2.1. Prokaryotic Expression and Identification of Recombinant Proteins

To screen for the optimal expression region of the 100K protein, the hydrophilicity of the amino acid sequence was predicted using the ExPASy-ProtScale online tool based on the Kyte-Doolittle method, with regions exhibiting high hydrophilicity scores prioritized. Antigenic epitopes of the protein were predicted using the NovoFocus tool. The three-dimensional structure of the 100K protein was predicted by AlphaFold3, and candidate regions identified through hydrophilicity and epitope prediction were mapped onto this spatial structure model to visually assess their surface accessibility. The region spanning amino acids 672 to 1053 (corresponding to nucleotides 2014 bp to 3162 bp of the *100K* gene) was ultimately selected, because it combines a relatively high hydrophilicity score, a high density of predicted antigenic epitopes, and favorable surface exposure characteristics in the structural model.

Primers for the amplification of the nonstructural protein genes *100K* and *22K* were designed with reference to the FAdV-4 GX2019-010 strain (GenBank accession number: MW439040). Restriction enzyme recognition sites were introduced at the 5′ ends of the primers (Table 1). The primers were synthesized by BGI Co., Ltd., Shenzhen, China. The amplified *100K* and *22K* gene fragments were ligated into the pET-32a (+) plasmid (Cat: 69015 M; Sigma, Kawasaki, Japan), resulting in a fusion protein with two His-tags. The recombinant plasmids were verified by double enzyme digestion (Cat: 1060A, 1040A, 1094A; Takara, Kusatsu, Shiga, Japan) and DNA sequencing (BGI Co., Ltd., China).

BL 21 (DE3) chemically competent cells (Cat: CD601-02; TransGen Biotech, Beijing, China) carrying the recombinant plasmids were grown in LB medium at 37 °C until the OD600 reached 0.6–0.8. Protein expression was induced with IPTG at final concentrations of 0, 0.2, 0.4, 0.6, 0.8 and 1 mmol/L for 2, 4, 6, 8, 10 and 12 h, respectively. The cell pellet was collected and lysed in RIPA buffer (Cat: R0010, Solarbio, Beijing, China) on ice for 20 min, followed by sonication at 50 Hz with 5 s pulses and 10 s intervals until the bacterial suspension became clear. The samples were then mixed with 5× SDS-PAGE loading buffer (Cat: WB2001, NCM Biotech, Newport, RI, USA), heated at 100 °C for 10 min to ensure complete denaturation, cooled on ice, briefly centrifuged, and analyzed by SDS–PAGE (using the precast SDS-PAGE Gel 4–15%; Cat: PG41510-S; Solarbio) to determine the optimal induction conditions. The cells were sonicated on ice until clarified and then centrifuged at 12,000× *g* for 2 min. The supernatant and pellet fractions were analyzed by SDS–PAGE to assess recombinant protein solubility.

The inclusion bodies of the 100K protein were solubilized using binding buffer composed of 20 mmol/L Tris-HCl (pH 7.9), 5 mmol/L imidazole, 0.5 mol/L NaCl, and 8 mol/L urea. The recombinant protein was purified using a His-tag protein purification kit (Cat: CW0893S, CW0894S, CWBIO, Taizhou, China). After purification, the 100K protein was placed into a dialysis bag and refolded in refolding buffer (1× PBS, 1 mmol/L EDTA, and a decreasing concentration of urea, pH 8.0). The purification efficiency was assessed by SDS-PAGE. Western blotting was performed to identify the recombinant proteins using FAdV-4-positive serum as the primary antibody and HRP-conjugated goat anti-chicken IgG (Cat: D110205; BBI) as the secondary antibody.

### 2.2. Development and Optimization of ELISA Conditions

The optimal ELISA conditions were determined on the basis of the criteria of an OD450 value close to 1.0 and the highest positive/negative (P/N) ratio. All optimization experiments were performed in duplicate, and the mean OD450 value was used for analysis. The optimal conditions were selected primarily on the basis of the P/N ratio. When multiple parameter combinations exhibited similar P/N ratios (within a 5% difference), further selection was performed by considering the OD450 value of the positive control (preferring the value closest to 1.0). Purified recombinant proteins were diluted to various concentrations for plate coating, and primary antibody (FAdV-4-positive serum) was serially diluted (1:50–1:400) to determine the optimal coating concentration and serum dilution. The coating conditions were optimized by incubation at 4 °C or 25 °C for 14 h and at 37 °C for 1, 2, or 3 h followed by 4 °C for 14 h. After coating, the wells were blocked with 5% skim milk for 30–120 min to optimize the blocking time, and the primary antibody incubation was similarly optimized over the same time range. HRP-conjugated goat anti-chicken IgG was tested at dilutions ranging from 1:2500 to 1:20,000 with incubation times of 30–120 min to optimize the secondary antibody conditions. Color development was assessed at 5–15 min to determine the optimal substrate incubation time.

### 2.3. Cut-Off Value Determination

An optimized ELISA protocol was used to test 100 FAdV-4-negative SPF chicken serum samples. The absorbance at OD450 was measured for each sample, and the mean ($\bar{x}$) and standard deviation (s) were calculated. The cutoff value was defined as $\bar{x}$ + 3 SD. Samples with OD450 values greater than or equal to the cutoff and a sample-to-negative (S/N) ratio >2 were considered positive [26]; otherwise, they were classified as negative.

### 2.4. Specificity and Repeatability Evaluation

To evaluate assay specificity, the two ELISA methods were tested using serum samples positive for Newcastle disease virus (NDV), infectious bursal disease virus (IBDV), infectious bronchitis virus (IBV), avian reovirus (ARV), and avian influenza virus subtypes H5 (AIV-H5) and H9 (AIV-H9), as well as sera from chickens immunized with the inactivated FAdV-4 vaccine and from 12 serotypes of FAdV-infected birds. SPF chicken serum samples were used as negative controls.

For the repeatability assessment, five FAdV-4-positive and five SPF chicken-negative serum samples were tested. Intrassay repeatability was evaluated by testing each sample in triplicate at three different time points using the same batch of antigen-coated plates. Inter-assay repeatability was assessed using plates prepared from three different batches, with all the samples tested simultaneously.

### 2.5. Validation of the Identification Test

To evaluate the ability of the 100K-ELISA and 22K-ELISA to differentiate between natural infection and vaccine-induced antibodies, three sets of serum samples were tested: sera collected at week 3 from 50 chickens experimentally infected with the FAdV-4 strain; sera collected at week 3 from 50 chickens immunized with the inactivated Fowl Adenovirus (FAdV) Group I, Serotype 4 vaccine [27]; and sera from 50 uninfected/unvaccinated control chickens.

To assess the clinical diagnostic capability of the two in-house ELISAs (100K-ELISA and 22K-ELISA), a field evaluation was conducted using a commercially available Fowl Adenovirus Group I Antibody Test Kit (FAdV-I-ELISA; Cat: CK132, BioChek, Ascot, UK) as the reference method. This reference kit uses inactivated Fowl Adenovirus Group I antigen as the coating material and is specifically designed to detect antibodies against Fowl Adenovirus Group I in chicken serum samples. The evaluation was carried out by testing 96 serum samples from vaccinated commercial chickens and 220 serum samples from nonvaccinated commercial chickens.

The detection results for the 220 nonvaccinated chicken sera obtained with the commercial FAdV-I-ELISA were used as the reference standard for statistical analysis of the data from the 100K-ELISA and 22K-ELISA. Diagnostic agreement was assessed by calculating the Kappa statistic (κ = (P_o_ − P_e_)/(1 − P_e_)). The clinical diagnostic value was evaluated by generating receiver operating characteristic (ROC) curves and calculating the area under the curve (AUC). The optimal cut-off value was determined by calculating the Youden Index (YI), and the corresponding diagnostic sensitivity and specificity at this cut-off are reported.

## 3. Results

### 3.1. Preparation and Characterization of Recombinant Proteins

**Figure 1:**
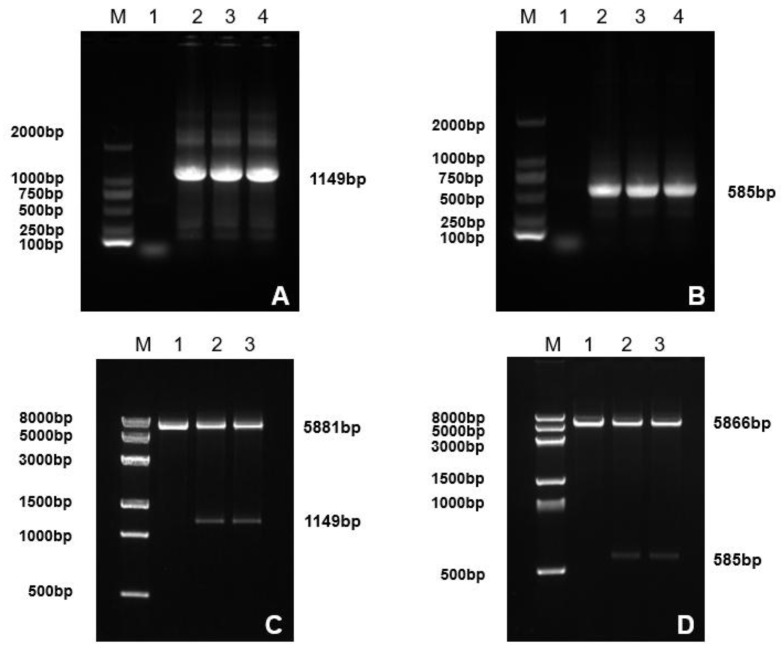
Amplification and identification of *100K* and *22K* genes. (**A**): M: DL2000 DNA marker; Lane 1: negative control; Lanes 2–4 show PCR products of the *100K* gene. (**B**): M: DL2000 DNA marker; Lane 1: negative control; Lanes 2–4 show the PCR products of the *22K* gene. (**C**): M: 8K DNA marker; Lane 1: pET-32a (+) plasmid; Lanes 2–3 shows the double digestion product of pET-32a-100K. (**D**): M: 8K DNA marker; Lane 1: pET-32a (+) plasmid; Lanes 2–3 show the double digestion product of pET-32a-22K.

PCR amplification produced 100K and 22K fragments of 1149 bp and 585 bp, respectively. Double digestion and sequencing confirmed successful insertion into the pET-32a (+) vector, yielding the recombinant plasmids pET-32a-100K and pET-32a-22K (Figure 1).

**Figure 2:**
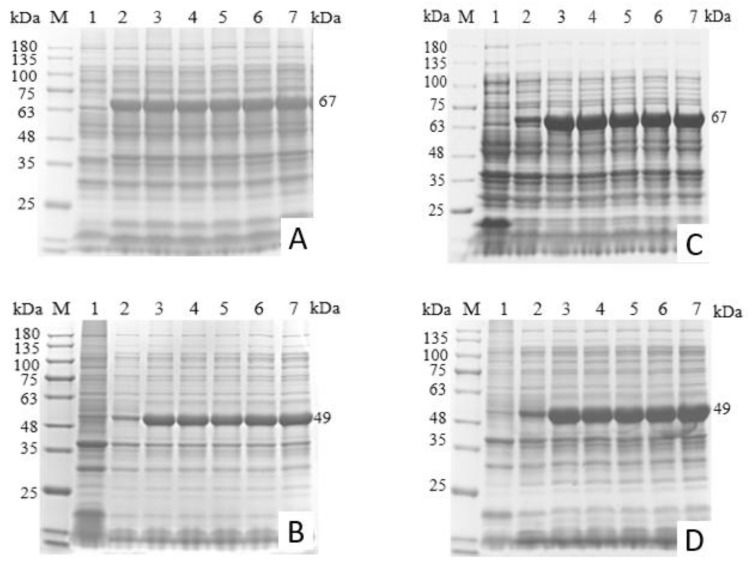
SDS–PAGE analysis of induction conditions for 100K and 22K proteins. (**A**,**B**): Expression of recombinant proteins from pET-32a-100K and pET-32a-22K at different induction times. M: ColorMixed protein marker (11–180KD); Lane 1: Empty vector expression control; Lanes 2–7: Samples induced for 2, 4, 6, 8, 10 and 12 h, respectively. (**C**,**D**): Expression of recombinant proteins from pET-32a-100K and pET-32a-22K induced with different IPTG concentrations. M: ColorMixed protein marker (11–180KD); Lane 1: Empty vector expression control; Lanes 2–7: Samples induced with IPTG at 0, 0.2, 0.4, 0.6, 0.8, and 1.0 mM, respectively.

Upon IPTG induction, the recombinant proteins were expressed at 67 kDa (100K) and 49 kDa (22K), which was consistent with the expected sizes. The expression levels of the recombinant proteins were similar across 0.2–1.0 mmol/L IPTG; 0.2 mmol/L for 4 h was selected (Figure 2).

**Figure 3:**
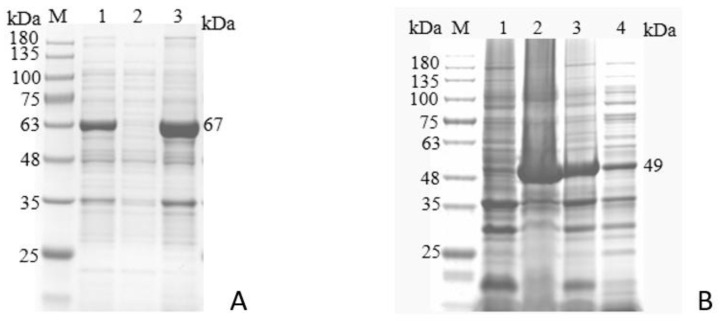
Solubility analysis of the recombinant proteins. (**A**): SDS–PAGE analysis of the pET-32a-100K recombinant protein. M: ColorMixed protein marker (11–180KD); Lane 1: Whole cell lysate; Lane 2: Soluble fraction of the induced culture; Lane 3: Insoluble fraction of the induced culture. (**B**): SDS–PAGE analysis of the pET-32a-22K recombinant protein. M: ColorMixed protein marker (11–180KD); Lane 1: Empty vector expression control; Lane 2: Whole cell lysate; Lane 3: Soluble fraction; Lane 4: Insoluble fraction.

SDS–PAGE of the soluble and insoluble fractions revealed that 100K was expressed mainly as inclusion bodies, whereas 22K was largely soluble (Figure 3).

**Figure 4:**
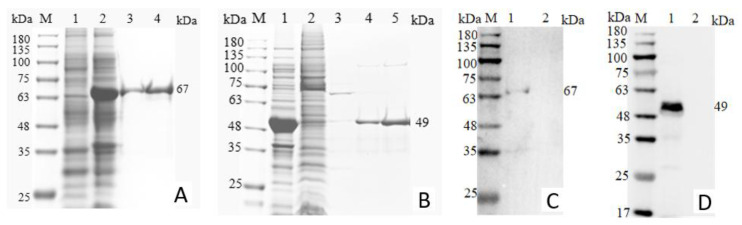
Purification and identification of recombinant proteins. (**A**): Purification of the pET-32a-100K recombinant protein. M: ColorMixed protein marker (11–180KD); Lane 1: Empty vector expression control; Lane 2: Crude lysate before purification; Lanes 3–4: Purified protein fractions. (**B**): Purification of the pET-32a-22K recombinant protein. M: ColorMixed protein marker (11–180KD); Lane 1: Crude lysate before purification; Lanes 2–3: Wash fractions; Lanes 4–5: Purified protein fractions. (**C**,**D**): Western blot analysis of pET-32a-100K and pET-32a-22K recombinant proteins. M: ColorMixed protein marker (11–180KD); Lane 1: recombinant protein; Lane 2: negative control.

Ni-affinity purification yielded single, specific bands for both proteins. Western blot analysis using FAdV-4-positive serum confirmed strong antigenic reactivity (Figure 4).

### 3.2. Optimization of ELISA Reaction Conditions

Through checkerboard titration experiments, the optimal coating concentrations of the recombinant proteins for the 100K-ELISA and 22K-ELISA methods were determined to be 6 μg/mL and 16 μg/mL, respectively, and the optimal dilution of the primary antibody serum was 1:100 for both methods. The optimal coating conditions for both methods involved incubation at 37 °C for 2 h, followed by further incubation at 4 °C for 14 h. The optimal blocking time using 5% skim milk was 60 min for both methods. The optimal incubation time for the primary antibody serum was 30 min. For both methods, the optimal dilution of the secondary antibody was 1:5000, and the optimal incubation time with the HRP-conjugated secondary antibody was 30 min. The optimal color development time was determined to be 10 min. The data related to the optimization are provided in Supplementary Materials.

### 3.3. Cut-Off Value of the ELISAs

Using the optimized ELISA protocol, OD450 values were measured for 100 serum samples collected from SPF chickens. The cutoff value for the 100K-ELISA was determined using the formula $\bar{x}$ + 3 SD, yielding a threshold of 0.346. Samples with OD450 values greater than or equal to 0.346 were considered positive, while those below this threshold were classified as negative. Following the same procedure, the cutoff value for the 22K-ELISA was calculated to be 0.382.

### 3.4. Specificity and Reproducibility of the ELISA Methods

**Figure 5:**
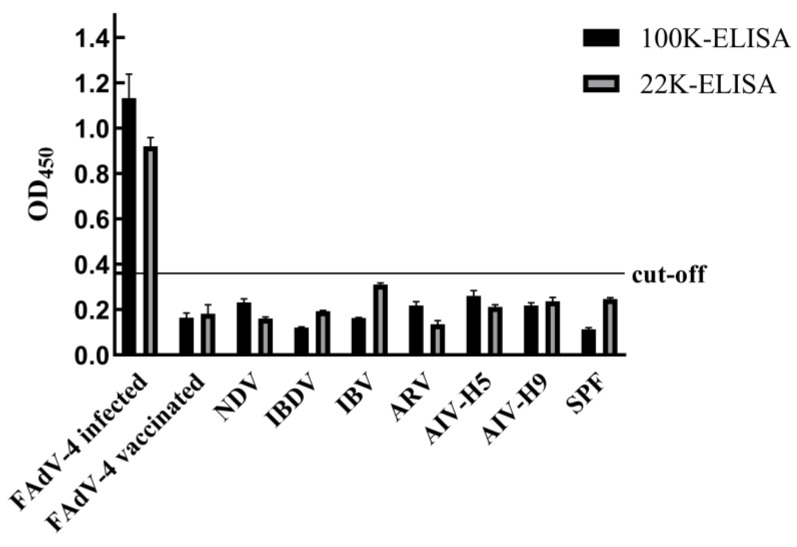
Specificity test results for different virus sera.

**Figure 6:**
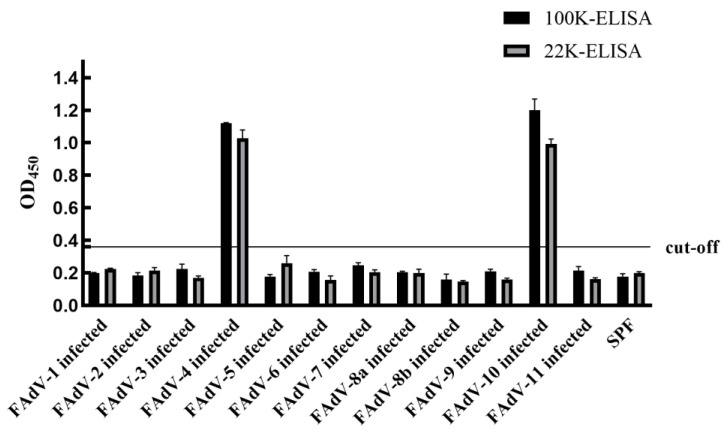
Specificity test results for different FAdV serotypes.

The two established ELISA methods were applied to serum samples from chickens experimentally infected with 12 serotypes of FAdVs, as well as serum samples from chickens immunized with an inactivated FAdV-4 vaccine. In addition, serum samples positive for other common avian pathogens, including NDV, IBDV, IBV, ARV, AIV-H5 and AIV-H9, were tested. Negative serum samples were included as controls. The results demonstrated that both ELISAs reacted specifically with serum samples from FAdV-4-infected and FAdV-10-infected chickens and showed no cross-reactivity with serum samples positive for other avian viruses or with serum samples from inactivated FAdV-4 vaccinated chickens. These findings indicate that both ELISA methods exhibit high specificity (Figure 5 and Figure 6).

To evaluate the intra-assay repeatability of the established ELISA methods, five positive and five negative serum samples were tested at three different time points using ELISA plates coated from the same batch. The coefficient of variation (CV) for the 100K-ELISA ranged from 2.0% to 4.5%, whereas that for the 22K-ELISA ranged from 1.7% to 4.2%, both of which were less than 5%, indicating good intrabatch repeatability for both assays (Table 2 and Table 3).

For interassay repeatability, the same samples were tested simultaneously using ELISA plates coated from three different batches. The results revealed that the CVs for both ELISA methods were less than 5%, demonstrating good interbatch consistency of the established assays (Table 2 and Table 3).

### 3.5. Sample Analysis

**Figure 7:**
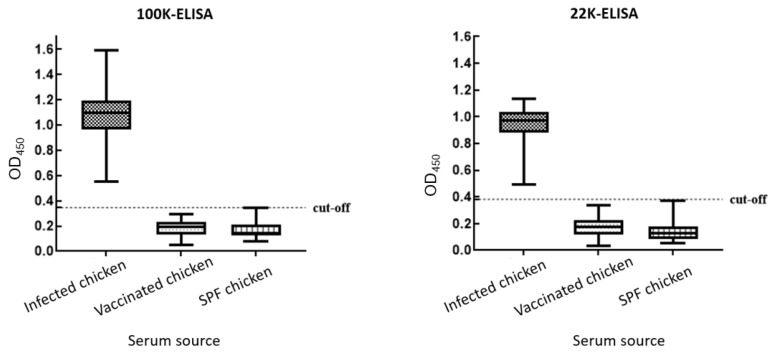
Results of the 100K-ELISA and 22K-ELISA testes.

A total of 150 serum samples from SPF chickens were tested using 100K-ELISA and 22K-ELISA, including 50 from FAdV-4-infected birds, 50 from chickens immunized with an inactivated FAdV-4 vaccine, and 50 from uninfected controls. Both ELISAs correctly identified all 50 infected samples as positive, while all sera from vaccinated and control birds tested negative. These results indicate that the developed ELISA methods can effectively differentiate between antibodies induced by FAdV-4 infection and those elicited by inactivated vaccine immunization (Figure 7).

The 100K-ELISA and 22K-ELISA methods were used to test clinical serum samples collected from chicken farms, including 96 samples from chickens vaccinated with the inactivated FAdV-4 vaccine and 220 samples from unvaccinated chickens. The results were compared with those obtained using a commercial FAdV-I-ELISA kit. In the vaccinated group, the positive detection rates were 100% for the FAdV-I-ELISA, 3.1% for the 100K-ELISA, and 5.2% for the 22K-ELISA. Among the 220 unvaccinated clinical samples, the positive rates were 21.8%, 18.6%, and 20.5% for the FAdV-I-ELISA, 100K-ELISA, and 22K-ELISA, respectively (Table 4 and Table 5).

Kappa value analysis revealed that the 100K-ELISA had a Kappa value of 0.902 (95% CI: 0.830–0.973), while the 22K-ELISA exhibited an even higher Kappa value of 0.959 (95% CI: 0.913–1.000), both indicating almost perfect agreement with the results of the FAdV-I ELISA. Notably, the 22K-ELISA produced fewer false-negative cases (3 vs. 7), demonstrating better detection capability for positive samples.

**Figure 8:**
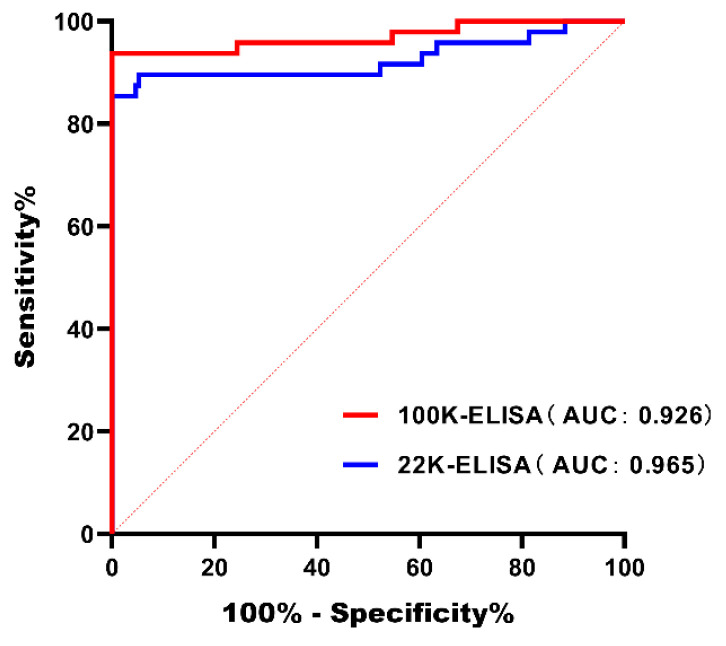
Diagnostic sensitivity and specificity of the ELISA kits as determined by ROC curve analysis.

The results of the ROC curve analysis are presented in Figure 8. The ROC curves of both ELISAs are located above and far from the diagonal reference line, with a steep rise at the initial portion of the curves, indicating the high resolution of the assays. The AUC values are shown in Table 6. According to the classification criteria proposed by Mandrekar [28], an AUC > 0.9 indicates high diagnostic accuracy. In this study, the AUC was 0.926 for the 100K-ELISA and 0.965 for the 22K-ELISA, demonstrating that both methods possess high diagnostic accuracy.

The optimal cut-off values and corresponding diagnostic performance were determined using the Youden index. For the 100K-ELISA, the Youden index was 0.8542, corresponding to a cut-off value of 0.3549, with a diagnostic sensitivity of 85.42% and a specificity of 100%. For the 22K-ELISA, the Youden index was 0.9375, corresponding to a cut-off value of 0.4136, with a diagnostic sensitivity of 93.75% and a specificity of 100%. These results indicate that both kits achieve highly specific detection results along with reliable sensitivity at their respective optimal cut-offs, supporting their utility as effective serological detection tools. (Table 6)

## 4. Discussion

FAdV-4 has been identified as the causative agent of the highly lethal avian disease HHS. Infected poultry experience rapid disease progression, with high mortality rates, typically resulting in death within 3 to 5 days post infection [29]. Clinically, infected chickens exhibit symptoms such as depression, ruffled feathers, decreased appetite, huddling in corners, and the excretion of greenish diarrhea [30]. Necropsy reveals typical lesions, including the presence of pale yellow fluid in the pericardial cavity and swelling and yellowing of the liver [31]. Histopathological examination of deceased chickens revealed intranuclear inclusions in liver cells, as well as degeneration and necrosis in the thymus, spleen, kidneys, and bursa of Fabricius [32]. In addition, the disease has multiple transmission routes, including horizontal transmission through food, feces, and farming equipment, as well as vertical transmission via infected chicken embryos [33]. These factors complicate the prevention and control of FAdV-4 infection [8]. Vaccination is a key strategy for preventing FAdV-4 infection. Currently, inactivated FAdV-4 vaccines are widely used on commercial poultry farms [34]. Therefore, developing an effective diagnostic method to differentiate between naturally infected chickens and those immunized with inactivated vaccines is crucial for flock sanitation and controlling the spread of FAdV-4 in poultry farms.

Nonstructural proteins (NSPs) are encoded by the viral genome and play essential roles in virus packaging and replication processes, but they do not form part of the mature virus particle. The main nonstructural proteins in avian adenoviruses include E1, E2, pol, 100K, 52/55K, 22K, 33K, and ADP (E3) [35,36]. The 100K and 22K proteins are important nonstructural proteins abundantly expressed during the late stage of adenovirus infection, which can effectively stimulate the host to produce antibody responses [37,38]. Although these proteins are encoded together with the 33K protein within the major late transcription unit L4 region, their coding sequences reside in unspliced mRNA regions [39]. From a genetic engineering perspective, the coding sequences of the 100K and 22K proteins contain no introns, facilitating their direct cloning into prokaryotic expression vectors and circumventing the obstacle that eukaryotic splicing machinery cannot function in prokaryotic systems. Therefore, considering the technical feasibility of prokaryotic expression, we selected the 100K and 22K proteins as candidate coating antigens for developing discriminatory ELISA methods.

Shah et al. [40] performed morphological characterization of a purified 100K protein using electron microscopy and reported that the protein exists as a trimer, forming a symmetric dumbbell-shaped molecule composed of two globular domains and a rod-like domain. The globular domains contain Hexon protein binding sites. When Hexon and 100K are coexpressed, the 100K protein acts as a molecular chaperone, facilitating the folding of Hexon trimers into soluble Hexon complexes, which are then transported to the cell nucleus [37,41]. The 100K protein also plays a crucial role in protecting the virus from host immune attacks. It interacts with Granzyme B (GrB), a cytotoxic lymphocyte granzyme that is essential for mediating cytotoxicity. This interaction is critical for the ability of a virus to evade immune responses and maintain its infectivity [42]. Prokaryotic expression of the full-length 100K protein is associated with a significant risk of misfolding. In this study, the 672–1053 fragment was selected for truncated expression to increase the probability of correctly folded proteins being obtained. The 22K protein is released only during viral replication. It plays a role in recognizing the transcriptional elements of the major late promoter (MLP) of AdV and interacts with the downstream elements within the MLP [43,44]. Morris et al. [45] reported that the 22K protein not only activates the expression of late genes but also suppresses the expression of early genes, thus regulating the timing of viral gene expression. Additionally, the 22K protein is essential for the binding of two other packaging proteins, IVa2 and L1-52/55K, to this region. The 22K protein interacts with IVa2, playing a key role in recognizing the adenovirus genome packaging domain, which enables the encapsidation of viral DNA [45]. Viruses with mutations in the 22K protein produce only empty viral capsids, which confirms that the 22K protein is essential for the packaging of the adenovirus genome [38].

The ELISA method established in this study, which is based on recombinant nonstructural proteins (100K and 22K), is designed not to directly detect the virus itself, but to trace the source of the immune response by detecting antibodies against FAdV-4 nonstructural proteins in chicken serum, thereby distinguishing natural infection from vaccination. This strategy aligns with the concept of DIVA (Differentiating Infected from Vaccinated Animals) assays. The interpretation of test results follows the logic of the “exclusion principle” to accurately identify infected chickens from the population, while the remaining negative individuals constitute a population that has not experienced natural infection (which includes vaccinated chickens).

To evaluate the effectiveness of the 100K-ELISA and 22K-ELISA methods in distinguishing between FAdV-4-infected chickens and those immunized with inactivated vaccines, this study used positive serum collected from SPF chickens at 3 weeks post-immunization with the FAdV-4 inactivated vaccine and artificially infected chickens. Neither of these methods detected antibodies in the serum of vaccinated chickens, but both methods successfully detected antibodies in the serum of infected chickens. These findings indicate that these methods can effectively differentiate between vaccine-induced and infection-induced antibodies, allowing the identification of FAdV-4 infection.

In the detection of 96 inactivated vaccine-immunized samples from a poultry farm, the 100K-ELISA and 22K-ELISA methods identified 3 and 5 samples, respectively, with low levels of antibody positivity. This suggests that although the flock was vaccinated, FAdV-4 infection may still be ongoing. When 220 nonimmunized chicken samples from the same farm were tested using 100K-ELISA and 22K-ELISA, the results were compared with those of a commercial FAdV-I-ELISA kit. These findings suggested that there may have been some level of FAdV-4 infection in the flock. Notably, antibody kinetics may have affect the long-term stability of DIVA performance. The absence of detection at multiple time points is indeed a limitation of this study. In future studies, we will collect serum samples at various time points postimmunization (e.g., 1, 2, 3, 4, and 5 weeks) for systematic testing to comprehensively assess the influence of antibody dynamics on the differential capability of the established ELISA method and to further validate its reliability in practical applications.

Regarding chickens that are both vaccinated and naturally infected, we recommend effectively identifying and managing such situations through the following approaches: For flocks with a known vaccination history (i.e., those that have been fully immunized with an inactivated vaccine), testing can be conducted using the 100K-ELISA or 22K-ELISA established in this study alone. A positive result indicates that although the chicken was vaccinated, a breakthrough infection may have occurred (i.e., the chicken is both vaccinated and infected). These individuals represent evidence of viral transmission under immune pressure and require focused attention. A negative result indicates that the chicken was only vaccinated and has not experienced infection. For flocks with unclear vaccination backgrounds, we recommend a combined testing strategy using a commercial whole virus ELISA together with our established ELISA: 1. A positive result for both 22K/100K-ELISA and the whole virus ELISA indicates that the chicken has experienced a natural infection. 2. A negative result for 22K/100K-ELISA but a positive result for the whole virus ELISA indicates that the chicken was only vaccinated and has not experienced infection. 3. A negative result for both 22K/100K-ELISA and the whole virus ELISA indicates that the chicken has neither been infected nor vaccinated.

Both ELISA methods were analyzed using ROC curves. According to the evaluation criteria proposed by Mandrekar [28], an AUC of 0.5 indicates no discriminatory ability, 0.7–0.8 is considered acceptable, 0.8–0.9 is considered excellent, and >0.9 is considered outstanding. In this study, the AUC was 0.926 for the 100K-ELISA and 0.965 for the 22K-ELISA, indicating that both methods possess high diagnostic accuracy. However, since the detection rates of the 100K-ELISA and 22K-ELISA were lower than those of the reference method, it is speculated that the commercial FAdV-I-ELISA—which is based on an inactivated whole-virus-coated ELISA—may have higher sensitivity because of the presence of more antibody binding sites in the serum. However, this inference is based solely on the method design rather than on direct comparative sensitivity experiments.

Notably, our initial calculation of the cut-off values, which was based on 100 SPF chicken negative serum samples using the conventional formula of mean + 3 × SD commonly employed in ELISA development, yielded values of 0.346 for the 100K ELISA and 0.382 for the 22K ELISA. Subsequently, ROC curve analysis was performed using a panel of well-defined serum samples (confirmed by a commercial FAdV group I ELISA kit), comprising 48 positive and 172 negative samples, all originating from non-vaccinated flocks. Based on the Youden’s index, the optimal cut-off values were determined to be 0.3549 for the 100K ELISA and 0.4136 for the 22K ELISA. The close agreement between these two sets of cut-off values was further corroborated when applied to all clinical samples (96 sera from vaccinated chickens and 220 field samples from non-vaccinated chickens), yielding identical positive/negative determinations. This consistency further confirms the reliability of the method established in this study. For practical clinical applications, we recommend using the cut-off values derived from the ROC analysis for two reasons: 1. These values are optimized based on empirical data from the actual target population, making them more suitable for real-world scenarios; 2. The cut-off values selected based on the Youden’s index achieve an optimal balance between sensitivity and specificity, which is further supported by their high area under the curve (AUC) values.

## 5. Conclusions

This study successfully developed two indirect ELISA methods, 100K-ELISA and 22K-ELISA, both of which demonstrated strong specificity and good repeatability. Both methods effectively distinguished between chickens immunized with the inactivated FAdV-4 vaccine and those that were naturally infected. These assays provide valuable technical support for the detection, monitoring, and control of FAdV-4 infections in poultry populations.

## Figures and Tables

**Table 1:** Sequences of specific primers.

Primer Name	Primer Sequences (5′ → 3 ′ )	Product Length	Digestion Site
100K-F	CCG*GAATTC*ATGGAGCGCAGTAACATTC	1149 bp	*Eco*R I
100K-R	CCC*AAGCTT*TTACCTCGGGGTGCTCGG		*Hin*d III
22K-F	CCG*GAATTC*ATGGCCCAGAGAATGGTCG	585 bp	*Eco*R I
22K-R	CCG*CTCGAG*CTAGCGTTGCGAGCCCTCGC		*Xho* I

**Table 2:** Repeatability test results of 100K-ELISA.

Sample ID	Intra-Assay Precision			Interassay Precision		
	$\bar{x}$	SD	CV/%	$\bar{x}$	SD	CV/%
1	1.11	0.028	2.5	1.08	0.045	4.2
2	1.13	0.044	3.9	1.18	0.051	4.3
3	1.12	0.022	2.0	1.16	0.052	4.5
4	1.14	0.045	3.9	1.11	0.047	4.2
5	1.06	0.047	4.4	1.08	0.032	3.0
6	0.19	0.005	2.6	0.20	0.010	4.9
7	0.21	0.009	4.3	0.20	0.005	2.4
8	0.19	0.005	2.6	0.21	0.005	2.4
9	0.16	0.007	4.5	0.15	0.007	4.3
10	0.21	0.005	2.5	0.20	0.007	3.5

**Table 3:** Repeatability test results of 22K-ELISA.

Sample ID	Intra-Assay Precision			Interassay Precision		
	$\bar{x}$	SD	CV/%	$\bar{x}$	SD	CV/%
1	0.97	0.041	4.2	0.96	0.017	1.8
2	0.98	0.025	2.6	0.96	0.031	3.2
3	0.98	0.037	3.8	0.94	0.044	4.7
4	1.09	0.029	2.2	1.08	0.034	3.1
5	0.85	0.021	2.5	0.86	0.026	3.0
6	0.22	0.006	2.7	0.23	0.008	3.5
7	0.21	0.004	1.7	0.22	0.005	2.3
8	0.29	0.005	1.9	0.28	0.009	3.4
9	0.22	0.009	4.2	0.22	0.010	4.5
10	0.25	0.009	3.6	0.24	0.009	3.8

**Table 4:** Test results of the clinical samples.

ELISA Methods	Vaccinated Group (n = 96)			Unvaccinated Group (n = 220)		
	Positive (n)	Negative (n)	Positive Rate (%)	Positive (n)	Negative (n)	Positive Rate (%)
FAdV-I-ELISA	96	0	100	48	172	21.8
100K-ELISA	3	93	3.1	41	179	18.6
22K-ELISA	5	91	5.2	45	175	20.5

**Table 5:** Comparison of the 2 × 2 contingency tables for the two in-house ELISA kits and the reference kit (n = 220).

ELISA Methods		FAdV-I-ELISA Positive (n = 48)	FAdV-I-ELISA Negative (n = 172)	Total
100K-ELISA	Positive	41 (TP)	0 (FP)	41
	Negative	7 (FN)	172 (TN)	179
22K-ELISA	Positive	45 (TP)	0 (FP)	45
	Negative	3 (FN)	172 (TN)	175

Note: TP: true positive; FP: false positive; FN: false negative; TN: true negative.

**Table 6:** Diagnostic performance of the ELISA methods based on ROC curve analysis.

ELISA Methods	AUC	Std. Error	95% Confidence Interval	Cut-Off	Sensitivity (%)	Specificity (%)
100K-ELISA	0.926	0.031	0.8646–0.9872	0.3549	85.42	100
22K-ELISA	0.965	0.018	0.9334–1.000	0.4136	93.75	100

## Data Availability

The original contributions presented in this study are included in the article/Supplementary Material. Further inquiries can be directed to the corresponding authors.

## Supplementary Materials

The following supporting information can be downloaded at: https://www.mdpi.com/article/10.3390/microorganisms14040842/s1, Table S1: The optimal coating concentration of the 100K recombinant antigen and the optimal serum dilution; Table S2: The optimal coating concentration of the 22K recombinant antigen and the optimal serum dilution; Table S3: Identification of the coating conditions; Table S4: Determination of the optimal blocking time; Table S5: Determination of the optimal serum incubation time; Table S6: Determination of the optimal dilution of the HRP-conjugated secondary antibody; Table S7: Determination of the optimal HRP-conjugated secondary antibody incubation time; Table S8: Determination of optimal color development time. The data related to the optimization of ELISA reaction conditions in this study are provided in Supplementary Materials.
