# Supplementary material for: Recombinant Protein-Based ELISA for the Detection and Differentiation of Antibodies Against Fowl Adenovirus Serotype 4 in Infected and Vaccinated Chickens
Source: Microorganisms. 2026 Apr 8;14(4):842. doi: 10.3390/microorganisms14040842 (PMC13118491; doi:10.3390/microorganisms14040842)
Supplement: Supplementary file 1 [file microorganisms-14-00842-s001.zip › microorganisms-4195089-supplementary.pdf]

Table S1 The optimal coating concentration of the 100K recombinant antigen and the optimal serum dilution.

Serum dilution		Coating concentration of the 100K recombinant antigen (μg/mL)				
		48	24	12	6	3
1:50	+	1.511	1.485	1.421	1.331	1.021
	-	0.234	0.208	0.174	0.146	0.104
	P/N ratio	6.5	7.1	8.2	9.1	9.8
1:100	+	1.269	1.200	1.003	**0.977**	0.654
	-	0.128	0.118	0.094	**0.077**	0.061
	P/N ratio	9.9	10.2	10.7	**12.7**	10.7
1:200	+	0.891	0.836	0.722	0.631	0.314
	-	0.084	0.078	0.066	0.057	0.045
	P/N ratio	10.6	10.7	10.9	11.1	7.0
1:400	+	0.539	0.518	0.459	0.377	0.201
	-	0.058	0.056	0.048	0.043	0.038
	P/N ratio	9.3	9.3	9.6	8.8	5.3

Table S2 The optimal coating concentration of the 22K recombinant antigen and the optimal serum dilution

Serum dilution		Coating concentration of the 22K recombinant antigen (μg/mL)				
		32	16	8	4	2
1:50	+	1.453	1.395	1.077	0.697	0.385
	-	0.281	0.238	0.161	0.113	0.059
	P/N ratio	5.2	5.9	6.7	6.2	6.5
1:100	+	1.127	**0.995**	0.701	0.479	0.245
	-	0.173	**0.135**	0.110	0.078	0.041
	P/N ratio	6.5	**7.4**	6.4	6.1	6.0
1:200	+	0.737	0.641	0.408	0.270	0.141
	-	0.109	0.093	0.060	0.041	0.029
	P/N ratio	6.8	6.9	6.8	6.6	4.9
1:400	+	0.426	0.358	0.237	0.156	0.081
	-	0.079	0.063	0.044	0.031	0.021
	P/N ratio	5.4	5.7	5.4	5.0	3.9

Table S3 Identification of the coating conditions

Coating condition	100K			22K		
	+	-	P/N ratio	+	-	P/N ratio

37 °C 1 h, then 4 °C 14 h	0.770	0.180	4.3	0.723	0.180	4.0
**37 °C 2 h, then 4 °C 14 h**	**1.020**	**0.190**	**5.4**	**0.830**	**0.196**	**4.2**
37 °C 3 h, then 4 °C 14 h	1.070	0.230	4.7	0.850	0.230	3.7
25°C 14h	1.030	0.200	5.2	0.797	0.206	3.9
4°C14h	0.760	0.180	4.2	0.624	0.190	3.3

Table S4 Determination of the optimal blocking time

Blocking time	100K			22K		
	+	-	P/N ratio	+	-	P/N ratio
30 min	0.890	0.180	4.9	0.990	0.229	4.3
**60 min**	**1.083**	**0.194**	**5.6**	**0.993**	**0.216**	**4.6**
90 min	1.069	0.218	4.9	0.955	0.221	4.3
120 min	1.016	0.226	4.5	0.854	0.213	4.0

Table S5 Determination of the optimal serum incubation time

Incubation time	100K			22K		
	+	-	P/N ratio	+	-	P/N ratio
**30 min**	**0.964**	**0.152**	**6.3**	**0.841**	**0.153**	**5.6**
60 min	1.082	0.201	5.4	1.040	0.234	4.4
90 min	1.134	0.228	5.0	1.127	0.259	4.4
120 min	1.197	0.260	4.6	1.151	0.294	3.9

Table S6 Determination of the optimal dilution of the HRP-conjugated secondary antibody

Secondary antibody dilution	100K			22K		
	+	-	P/N ratio	+	-	P/N ratio
1:2 500	0.986	0.226	4.4	0.990	0.270	3.7
**1:5 000**	**0.930**	**0.160**	**5.8**	**0.854**	**0.198**	**4.3**
1:7 500	0.680	0.130	5.2	0.621	0.159	3.9
1:10 000	0.550	0.118	4.7	0.387	0.126	3.1
1:15 000	0.470	0.100	4.7	0.270	0.088	3.1
1:20 000	0.360	0.080	4.5	0.234	0.069	3.4

Table S7 Determination of the optimal HRP-conjugated secondary antibody incubation time

Incubation time	100K			22K		
	+	-	P/N ratio	+	-	P/N ratio
**30 min**	**0.950**	**0.154**	**6.2**	**0.751**	**0.148**	**5.1**
60 min	1.180	0.210	5.6	0.978	0.230	4.3
90 min	1.234	0.233	5.3	1.055	0.270	3.9
120 min	1.229	0.243	5.1	1.031	0.299	3.5

Table S8 Determination of optimal color development time

Color development time	100K			22K		
	+	-	P/N ratio	+	-	P/N ratio
5 min	0.362	0.087	4.2	0.313	0.086	3.6
**10 min**	**1.024**	**0.162**	**6.3**	**0.810**	**0.144**	**5.6**
15 min	1.216	0.274	4.4	1.051	0.234	4.5
